# A Case of Gradenigo’s Syndrome in an Elderly Patient

**DOI:** 10.7759/cureus.63062

**Published:** 2024-06-24

**Authors:** Heabah Assi, Raul Alba, Mariam Hassan, John Demis, Thwe Htay

**Affiliations:** 1 Medicine, Texas Tech University Health Sciences Center Paul L. Foster School of Medicine, El Paso, USA; 2 Internal Medicine, Texas Tech University Health Sciences Center El Paso Paul L. Foster School of Medicine, El Paso, USA; 3 Internal Medicine, Texas Tech University Health Sciences Center El Paso, El Paso, USA; 4 Internal Medicine, Duke University Hospital, Durham, USA

**Keywords:** chronic otitis media, facial pain, petrous apicitis, gradenigo's syndrome, otitis media

## Abstract

Gradenigo's syndrome (GS) presents with the classical triad of otitis media, facial pain, and abducens nerve palsy as a complication of petrous apicitis. However, in the era of increased antibiotic use, complications of petrous apicitis have become infrequent and cases of GS are not frequently seen in clinical practice. We present the case of a 76-year-old man with uncontrolled diabetes mellitus presented with a two-month history of worsening right-sided headache, right-sided facial pain and weakness, along with dysphagia, hearing loss, and right otalgia with intermittent otorrhea following a right upper molar extraction. Imaging identified the inflammatory changes and indications of petrous apicitis. Although GS has become quite rare in recent years, this case highlights the importance of the responsible use of antibiotics in treating a seemingly innocuous infection.

## Introduction

Gradenigo's syndrome (GS) is diagnosed by the triad of otitis media, pain in the distribution of the ophthalmic and maxillary branches of the trigeminal nerve, and abducens nerve (cranial nerve six) palsy [1]. It is caused by the extension of an infection, typically an otitis media, to the adjacent petrous apex of the temporal bone [1], neoplastic lesions and tuberculous apicitis have been appreciated to cause GS [1-3]. There are many key structures in this region, namely the trigeminal ganglion and the abducens nerve, which are solely separated from the petrous bone by the dura mater and can be impinged with inflammation [4]. Thus, a patient will complain of facial pain and may experience horizontal diplopia secondary to unilateral esotropia.

This case was first presented at the American College of Physicians (Texas Chapter) Annual Scientific Meeting in November 2015. This case was also presented second time at the Annual Meeting of the Society of General Internal Medicine, Hollywood, FL, in May 2016.

## Case presentation

 A 76-year-old man with uncontrolled diabetes mellitus presented with a two-month history of worsening right-sided headache, right-sided facial pain, and weakness, along with dysphagia, hearing loss, and right otalgia with intermittent otorrhea following a right upper molar extraction. A CT scan done one week prior as a part of the facial pain investigation showed right sphenoid sinusitis and lucency in the molar extraction region. The physical examination findings were confined to the right side: mastoid tenderness, purulent discharge from the external auditory canal, profound mixed conductive and sensorineural hearing loss, lower motor neuron-type facial palsy, and rightward gaze diplopia.

**Figure 1:**
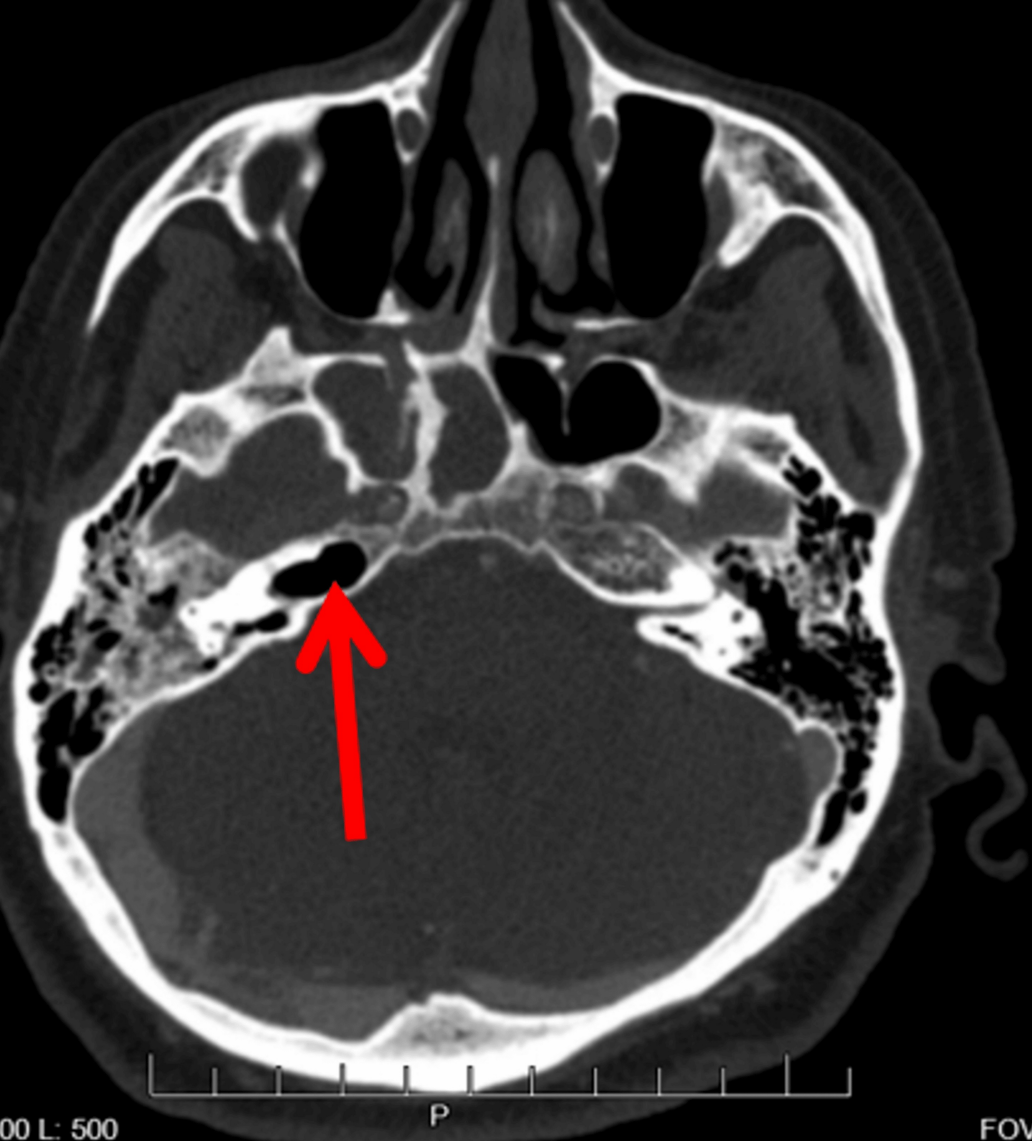
Initial CT showing osseous erosion of the right petrous temporal bone which also involves right carotid canal Red Arrow: Osseous Erosion

**Figure 2:**
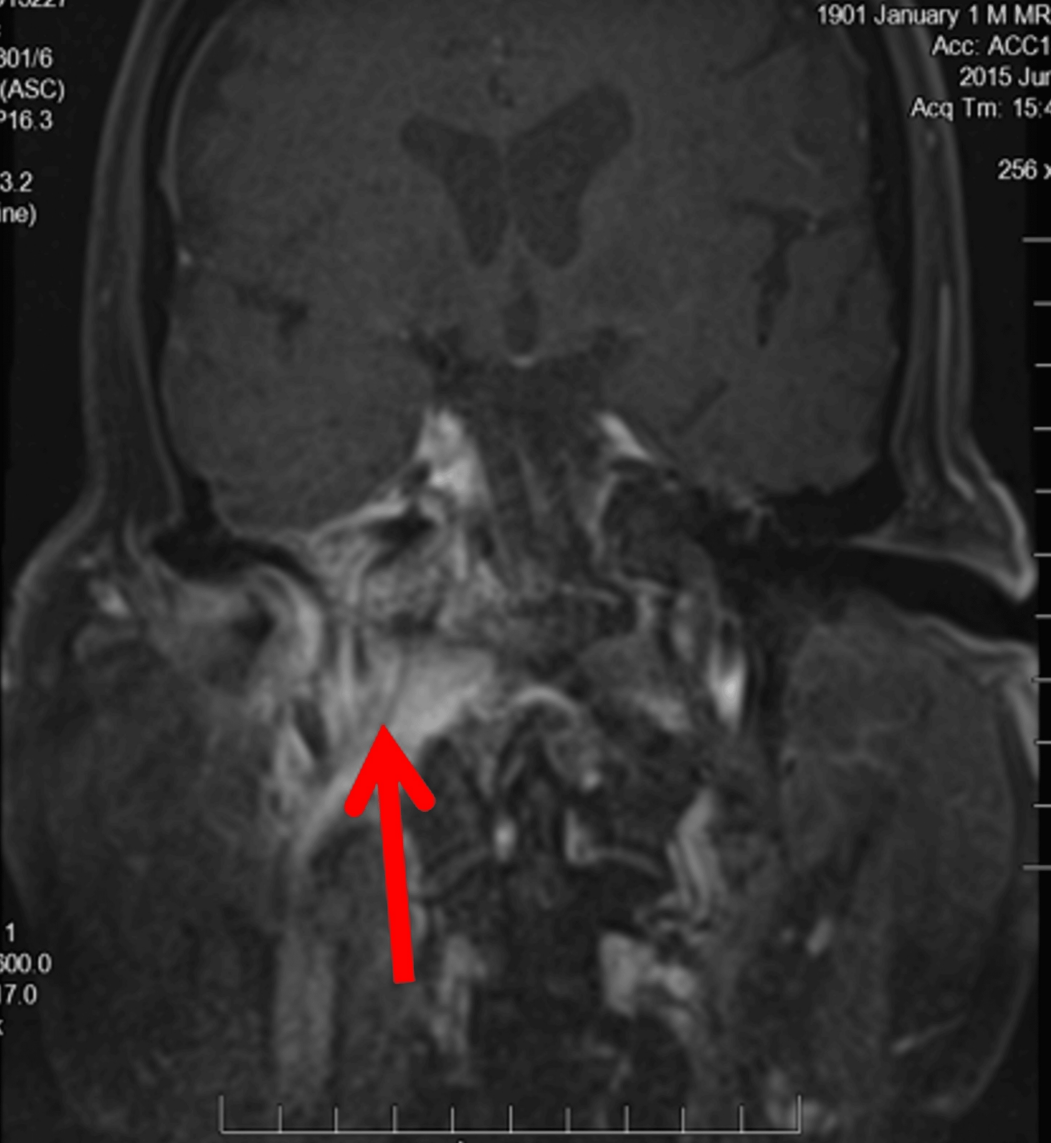
T1-weighted image showing enhancing soft tissue and inflammation near the petrous temporal bone Red Arrow: Enhancing soft tissue and inflammation

A CT (Figure 1) and an MRI (Figure 2) showed extensive inflammatory changes throughout the right petrous temporal bone, sphenoid sinus, right carotid space, right pterygopalatine fossa, and clivus. The three-phase technetium scan of the temporal bone showed increased focal uptake in the right mastoid region and petrous temporal bone consistent with right mastoiditis and petrous apicitis. The patient underwent a wide-field myringotomy and was discharged after several days of antibiotic treatment with ciprofloxacin-dexamethasone ear drops and a five to six-week course of intravenous cefepime and vancomycin.

**Figure 3:**
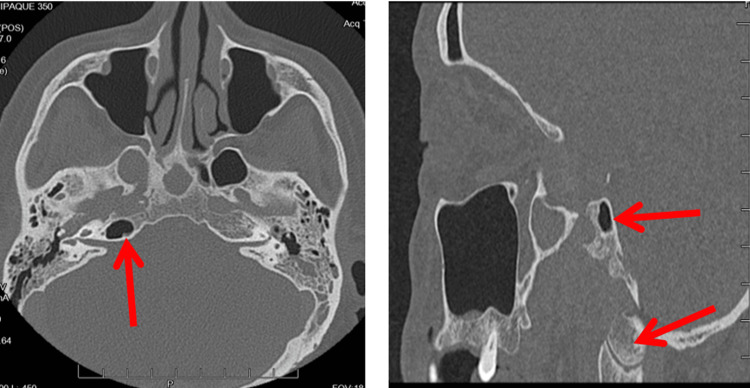
(Left and right) Repeat CT showing continued osseous erosion of the right petrous temporal bone now spreading to the clivus and right occipital condyle. Red Arrows: osseous erosion

Six weeks later, imaging studies were repeated and showed worsening osteomyelitis involving the right occipital condyle and left occipital bone (Figure 3).

His right-sided frontalis weakness had resolved, and dysphagia had improved, but the headache and otorrhea had become worse. He was readmitted and underwent right mastoidectomy, sphenoidotomy, and ethmoidectomy. Purulent fluid was cultured from the right sphenoid sinus and mastoid sinus, which showed growth of *Enterococcus *species and *Aspergillus* fumigatus. The patient was subsequently treated with a multidrug regimen of vancomycin, cefepime, metronidazole, and voriconazole, along with ciprofloxacin-dexamethasone ear drops.

## Discussion

GS was first described by Giuseppe Gradenigo in 1904 [2]. Patients were noted to present with initial complaints of otorrhea and otalgia, that preceded more concerning complications, such as diplopia and facial pain [2]. These physical exam findings are due to the neural and vascular structures located in this area, presenting with a classic triad of symptoms of otorrhea, pain in the trigeminal nerve distribution, and abducens nerve palsy [1]. An extraocular examination can reveal diplopia and lateral gaze palsy due to cranial nerve VI involvement [1]. Thus, clinicians must remain vigilant in the treatment of middle ear conditions given their capacity to evolve. Despite the infamous triad, in Gradenigo’s original study which comprised 57 patients, less than half presented with the complete triad, with some showcasing two out of three symptoms [3]. In more recent studies, 8 patients diagnosed with petrous apicitis did not present with the classic triad [4].

Several risk factors are associated with increased risk for the development of GS, such as patients with extensively pneumatized petrous apices. Petrous apicitis can be categorized into acute and chronic forms [4]. Acute petrous apicitis is characterized by the rapid development of symptoms due to abscess formation in a well-pneumatized apex, while chronic apicitis is a complication due to chronic otitis media with longstanding purulent otorrhea [4]. Due to the numerous structures in this area, the continuous spread of petrous apicitis to nearby structures leads to GS [1]. The commonly implicated organisms are those causing middle ear infections, such as *Staphylococcus aureus*, *Streptococcus pneumoniae*, *Haemophilus influenzae*, and *Pseudomonas*, while tuberculosis and fungal infection are less likely to be causes of infection [4]. Other factors that can increase the chance of this occurring include diabetics, the use of high-dose steroids, immunosuppressed individuals, and patients with cholesteatomas or chronic osteomyelitis who are susceptible to developing GS [5]. 

Petrous apicitis is most commonly a complication of otitis media infections, and less often appreciated in the setting of neoplastic lesions and fungal infections [1,3,6]. Cases of *Aspergillus *petrous apicitis have been documented, with overall mortality of *Aspergillus* osteomyelitis of the skull at about 27% [5]. In cases of *Aspergillus* petrous apicitis, clinical symptoms are often non-specific and can lead to diagnostic delay [6]. Involvement of the petrous apex is thought to be due to the movement of the fungus through air conduits between the apex and the base of the petrous bone [7].

The diagnostic imaging modality of choice to identify petrous apicitis is CT, given its sensitivity and low false positive rate [4]. The CT provides detailed imaging and can identify any lesions present, which in the case of petrous apicitis presents as lesions in the petrous apex [4]. Another method for diagnosis of petrous apicitis includes MRI, which can delineate between soft tissue and can further be used to evaluate lesions of the petrous apex [4]. Acute and chronic petrous apicitis appear differently on MRI and can help identify the potential underlying cause as if left untreated, GS can be fatal [4].

Prevention of this complication can be accomplished with adequate treatment of a new or ongoing infection with the appropriate antibiotic therapy and emphasizing to patients the importance of adherence to therapy. Nevertheless, the rampant usage of antibiotics has resulted in the almost complete disappearance of complications of petrous apicitis, such as GS [2]. If antibiotic treatment is not sufficient, surgery can be used [1].

## Conclusions

In cases of persistent unilateral facial pain and neurological signs, clinicians should have a high clinical suspicion for the development of petrous apicitis, a rare and serious complication distinguished by infection and inflammation of the temporal bone, and possibly GS, which manifests as the clinical triad of the trigeminal nerve, abducens nerve palsy, and otitis media. Although GS has been quite rare in recent years, this case showcases the importance of responsible use of antibiotics in treating a seemingly innocuous infection that can progress to GS.

## References

[1] Bano S, Nawaz A, Asmar A (2022). Gradenigo's syndrome presenting as IX and X cranial nerve palsy without clinically apparent ear infection: a case report and review of literature. eNeurologicalSci.

[2] Felisati D, Sperati G (2009). Gradenigo's syndrome and Dorello's canal. Acta Otorhinolaryngol Ital.

[3] Pedroso JL, de Aquino CC, Abrahão A (2011). Gradenigo’s syndrome: beyond the classical triad of diplopia, facial pain and otorrhea. Case Rep Neurol.

[4] Sherman SC, Buchanan A (2004). Gradenigo syndrome: a case report and review of a rare complication of otitis media. J Emerg Med.

[5] Stodulski D, Kowalska B, Stankiewicz C (2006). Otogenic skull base osteomyelitis caused by invasive fungal infection. Eur Arch Otorhinolaryngol.

[6] Bhatt Y, Pahade N, Nair B (2013). Aspergillus petrous apicitis associated with cerebral and peritubular abscesses in an immunocompetent man. J Laryngol Otol.

[7] Rushton P (2004). Aspergillosis of the petrous apex. Age Ageing.

